# Detection of circulating norovirus genotypes: hitting a moving target

**DOI:** 10.1186/1743-422X-11-129

**Published:** 2014-07-18

**Authors:** Brenda-Lee Rooney, Janice Pettipas, Elsie Grudeski, Oksana Mykytczuk, Xiao-Li Pang, Tim F Booth, Todd F Hatchette, Jason J LeBlanc

**Affiliations:** 1 Dalhousie University, Halifax, Nova Scotia, Canada; 2 Division of Microbiology, Department of Pathology and Laboratory Medicine, Capital District Health Authority (CDHA), Dalhousie University, Halifax, Nova Scotia, Canada; 3 Enteroviruses and Enteric Viruses Laboratory, National Microbiology Laboratory (NML), Winnipeg, Manitoba, Canada; 4 Food Virology Reference Centre, Bureau of Microbial Hazards, Health Canada, Ottawa, Ontario, Canada; 5 Provincial Laboratory for Public Health (ProvLab), Edmonton, Alberta, Canada; 6 Department of Laboratory Medicine and Pathology, University of Alberta, Edmonton, Alberta, Canada

**Keywords:** Norovirus, Proficiency testing, Quantitative RT-PCR, Epidemiology, Genotyping

## Abstract

**Background:**

Although national surveillance programs are in place to monitor norovirus epidemiology, the emergence of new strains and the genetic diversity among genotypes can be challenging for clinical laboratories. This study evaluated the analytical and clinical performance characteristics of one real-time RT-PCR and two end-point RT-PCRs commonly used in microbiology laboratories.

**Methods:**

Lower limit of detection (LoD) was determined using 10-fold dilutions of noroviruses belonging to different genotypes. The clinical performance of the real-time and end-point RT-PCRs was assessed in parallel using nucleic acids extracted from 186 stool specimens.

**Results:**

The real-time RT-PCR was highly sensitive and specific for the detection of norovirus genotypes that are currently circulating in Canada. In contrast, the two end-point RT-PCRs displayed poor analytical sensitivity or complete failure to detect certain norovirus genotypes, which was correlated to sequence mismatches in the primer-binding sites. In an attempt to improve norovirus detection with the end-point RT-PCRs, both assays were processed concurrently and detection from either assay was considered a positive result. Concurrent testing resulted in only a modest increase in clinical sensitivity (75.0%) compared to each assay alone (62.5% and 71.9%). However, the false positivity rate increased from 1.98% and 3.36% for the assays alone to 5.47% with concurrent testing.

**Conclusions:**

This study emphasizes the benefits of a real-time method and provides support for routine surveillance to monitor norovirus epidemiology and ongoing proficiency testing to ensure detection of circulating norovirus genotypes.

## Background

Noroviruses are the leading cause of acute gastroenteritis, and outbreaks are common [1,2]. Transmission occurs through the fecal-oral route and is facilitated by a low infectious dose and environmental persistence [1,2]. Laboratory identification of norovirus can help reduce transmission through infection control and public health interventions [2]. Since human noroviruses are uncultivable, traditional detection methods relied primarily on electron microscopy and enzyme immunoassays, both of which lack sensitivity [2-5]. RT-PCR has markedly improved the detection of noroviruses and has become the method of choice for clinical diagnosis [2]. However, the genetic diversity among noroviruses poses a particular challenge for molecular assays [6-11]. Noroviruses are classified into six genogroups, three of which cause human disease (GI, GII, and GIV) [12-14]. The two predominant genogroups, GI and GII, are further subdivided into 9 and 22 genotypes, respectively [12-14]. Strategies used to overcome norovirus diversity have included the simultaneous use of various monoplex RT-PCRs, multiplexed RT-PCRs, RT-PCRs with degenerate primers and probes [8-11,15-19].

The dynamic nature of its epidemiology poses further challenges for laboratory detection of norovirus. While genotype GII.4 is responsible for the majority of outbreaks annually, new GII.4 strains emerge every 2–3 years that replace the previously circulating pandemic strain [14,20-25]. Norovirus GII.4-2012 Sydney has recently emerged and replaced GII.4-2009 New Orleans worldwide [14,20-26], including the Canadian provinces of Alberta [25], British Columbia [27] and recently, Nova Scotia (unpublished data). In addition, the proportion of outbreaks associated with non-GII.4 genotypes has increased in Canada and the US, and the predominant non-GII.4 genotypes change over time [14,23,25,28]. With the diversity among norovirus genotypes and the dynamic nature of its epidemiology, this study evaluated the analytical and clinical performance of a real-time RT-PCR and two end-point RT-PCRs (EP-SR and EP-JV) commonly used for the detection of noroviruses in clinical, food and environmental laboratories [8-11,16,17,29-36].

## Results

### Analytical sensitivity and specificity

Each method was specific for circulating noroviruses (Table 1) and no cross-reactions occurred with various enteric pathogens. The real-time RT-PCR was able to detect all norovirus genotypes with high sensitivity (Table 1). EP-JV detected all GII.4 strains, but only a subset of non-GII.4 genotypes. EP-SR only detected GII.4 strains. For GII.4 noroviruses, real-time RT-PCR was approximately 10-fold more sensitive than EP-JV, and 100-fold more sensitive than EP-SR (Table 1). Similarly, the LoD for different GII.4 strains were highly consistent for EP-JV. For EP-SR, only minor differences in the LoD for GII.4 strains from 2004 to 2009, but a 100-fold decrease in sensitivity was observed for GII.4-2012. Reduced sensitivity was also observed with EP-JV for genotypes GI.6 and GII.1.

**Table 1:** Limit of detection and specificity analysis

**Genotype**	**Estimated analytical sensitivity [Log**_**10 **_**(copies/ml)]**		
	**Real-time**	**EP-JV**	**EP-SR**
	**RT-PCR**	**RT-PCR**	**RT-PCR**
Norovirus, genotype GI			
GI.1 (2011)	2.21	ND*	ND
GI.3 (2008)	2.27	ND	ND
GI.4 (2008)	2.67	ND	ND
GI.6 (2010)	2.11	5.96	ND
Norovirus, genotype GII (non-GII.4)			
GII.1 (2011)	2.37	5.54	ND
GII.3 (2006)	2.52	ND	ND
GII.7 (2010)	2.57	ND	ND
GII.13 (2010)	2.14	ND	ND
Norovirus, genotype GII.4			
GII.4 (2004) Den Haag	2.20	3.56	4.55
GII.4 (2006b) Osaka	2.47	3.37	4.32
GII.4 (2009) New Orleans	2.85	3.53	4.52
GII.4 (2012) Sydney	2.61	3.71	6.62

*ND signifies not detected at a minimal concentration of 10^6^ copies/ml.

### Clinical evaluation

**Figure 1:**
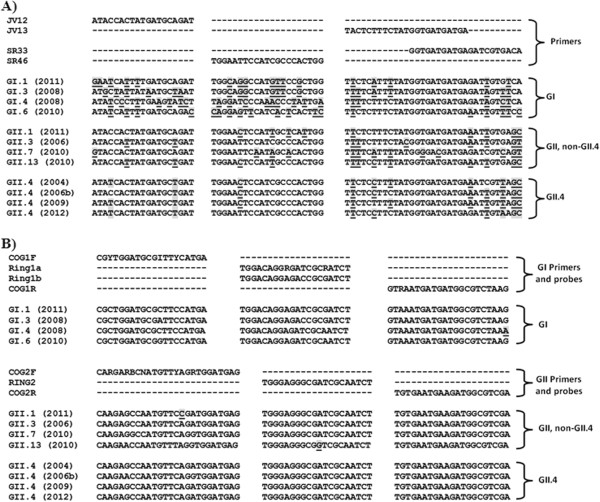
**Sequence alignment of the primer and probe binding sites.** Primer pairs JV12/JV13 and SR33/SR46 are **A)** endpoint RT-PCR assays; **B)** real-time RT-PCR. Mismatches between the primer/probe sequences are indicated by a shaded font and are underlined. Genbank accession numbers are as follows: GI.1 (2011), KF039733.1; GI.3 (2008), JN603244.1; GI.4 (2008), JN603245.1; GI.6 (2010), JQ388274.1; GII.1 (2011), KC597150.1; GII.3 (2006), GU980585.1; GII.7 (2010), JQ750988.1; GII.13 (2010), JX439807.1; GII.4 (2004), JQ798158.1; GII.4 (2006b), AB447442.1; GII.4 (2009), GU445325.2; GII.4 (2012), JX459908.1. Abbreviations: B = C, G, or T; R = A or G; Y = C or T; I = A, C, G, or T.

The clinical sensitivity of the real-time RT-PCR and end-point RT-PCR assays (EP-JV and EP-SR) was 100%, 71.9%, and 62.5%, respectively (Table 2). With concurrent testing of EP-JV and EP-SR, the clinical sensitivity was only modestly increased to 75% compared to each assay alone, since both EP-JV and EP-SR failed to detect 8 genotype GII.7 noroviruses (Table 3). EP-JV alone also missed a genotype GII.15 and EP-SR missed three genotype GI.6 noroviruses and one GI.3 (Table 3). With the exception of the latter, viral loads were above their LoD for all methods and the poor sensitivity of EP-JV and EP-SR was correlated to sequence mismatches in the primer-binding sites (Table 1 and Figure 1).

**Table 2:** Clinical performance characteristics compared to the modified gold standard

**Method**	Clinical Parameters*			
	**Sensitivity (%)**	**Specificity (%)**	**Kappa**	**False positivity rate (%)**
Real-time RT-PCR	100.0 (91.5 – 100.0)	100.0 (98.2 – 100.0)	1.00 (0.90 – 1.00)	0.00
EP-JV	71.9 (58.6 – 71.9)	98.1 (95.3 – 99.5)	0.76 (0.58 – 0.84)	1.99
EP-SR	62.5 (48.4 – 71.7)	96.8 (93.8 – 98.7)	0.65 (0.46 – 0.77)	3.36
EP-JV and EP-SR	75.0 (60.3 – 85.5)	94.8 (91.7 – 97.0)	0.70 (0.52 – 0.82)	5.47

*When applicable, 95% confidence intervals are indicated in parentheses.

**Table 3:** Discordant results obtained during the clinical evaluation

**No.**	**RT-PCR results**			**Discordant analysis**		**Interpretation**	**Comments**
	**Real-time RT-PCR**	**EP-JV**	**EP-SR**	**Result**	**Genotype**		
5	Neg.	Neg.	Pos.	Neg.	N/A	FP (EP-SR)	Non-specific amplification
3	Neg.	Pos.	Neg.	Neg.	N/A	FP (EP-JV)	Non-specific amplification
8	Pos.	Neg.	Neg.	Pos.	GII.7	FN (EP-JV and EP-SR)	High viral loads (5.28 to 7.78 log_10_ copies/ml)
3	Pos.	Pos.	Neg.	Pos.	GI.6	FN (EP-SR)	High viral loads (5.29 to 5.74 log_10_ copies/ml)
1	Pos.	Pos.	Neg.	Pos.	GI.3	FN (EP-SR)	Low viral load (2.47 log_10_ copies/ml)
1	Pos.	Neg.	Pos.	Pos.	GII.15	FN (EP-JV)	High viral load (6.33 log_10_ copies/ml)

*Abbreviations*: *FN* false negative, *FP* false positive, *N/A* not applicable, *Neg.* Negative, *No.* number, *Pos.* positive.

As non-specific amplifications were observed for both EP-JV and EP-SR, concurrent testing resulted in a reduced clinical specificity (94.8%) compared to each method alone (98.1% and 96.8%, respectively) (Table 2). The false positivity rate for concurrent testing was increased to 5.47% compared to 1.98% and 3.36% for EP-JV and EP-SR alone. A two-year retrospective analysis revealed that 14 outbreaks were declared by a single EP-JV and EP-SR result where weak amplifications were noted, suggesting a possible false positivity rate of 4.8% for years 2011 and 2012 (Additional file 1: Table S2). As no differences were observed between real-time RT-PCR and the reference methods, the real-time RT-PCR was highly specific (100%) and no false positives were observed (Tables 2 and 3).

## Discussion

Genomic diversity and evolutionary change can be challenging for detection of noroviruses [2,37,38]. While detection of noroviruses using RT-PCR is far more accurate than antigen-based detection methods (i.e. EIAs), not all molecular methods are created equal [2-4]. Fortunately, most reference laboratories use a real-time RT-PCR targeting the most conserved region of the genome (ORF1-ORF2 junction) and use degenerate primers and probes that can tolerate some sequence mismatches [9,10] (Figure 1). In this study, this same real-time RT-PCR was able to detect diverse norovirus genotypes with analytical sensitivities consistent with values previously reported for GII.4 (2006b) (Table 1) [6]. In contrast, EP-JV and EP-SR were far less sensitive and failed to detect certain genotypes (Tables 1, 2 and 3). With hopes to enhance detection of circulating norovirus genotypes, EP-JV and EP-SR were processed concurrently and detection from either assay was considered a positive result. Concurrent testing only modestly improved clinical sensitivity (Table 2) since the majority of false negative results were common between both EP-JV and EP-SR (Table 3). Interestingly, failure of EP-JV and EP-SR to detect certain genotypes was not attributed to poor analytical sensitivity since the viral load in most clinical specimens far exceeded the LoD for each assay (Tables 1 and 3). Instead, the genotypes that failed to be detected for EP-JV (GII.7, GII.15) and EP-SR (GII.7, GI.6, and GI.3) were linked to several sequence mismatches in the primer-binding sites (Figure 1). Of note, a large norovirus outbreak in Sweden was missed due to mismatches in the primer-binding sites of JV12 and JV13 (same used for EP-JV), but were detected by another primer pair [31].

It should also be noted that real-time RT-PCR and EP-JV both detected genotype GI.3 in a specimen with a very low viral load (2.47 log_10_ copies/ml), whereas EP-JV were unable to detect this genotype at concentrations exceeding 6.0 log_10_ copies/ml in the specificity analysis (Tables 1 and 3). This confounding result might be explained by genetic differences between the norovirus GI.3 identified in the clinical evaluation and the one used for the specificity analysis. A 12% nucleotide difference was noted between GI.6 and GI.7 genotypes that circulated in Canada between 2009 and 2010 compared to 2012 and 2013, suggesting that diversity among the same genotype can occur over time [24]. It is also possible that the faint amplicon detected by EP-JV was a false positive result in a specimen that was coincidently positive by real-time RT-PCR (Table 3).

While concurrent testing marginally increased the clinical sensitivity, a reciprocal effect was seen on the clinical specificity where the false positivity rate was higher with concurrent testing (5.47%) than either method alone (1.98% and 3.36% for EP-JV and EP-SR, respectively) (Table 2). Upon review of the false positive results, only faint or non-specific amplifications were noted and likely attributed to the low annealing temperature of EP-JV (37°C) or lack of electrophoretic resolution between primer dimmers and the small amplicon generated with EP-SR of 123 bp. The subjectivity of gel resolution is not a problem for a properly validated real-time RT-PCR [9,10,39].

Following a two-year retrospective analysis of EP-JV and EP-SR results, 14 of 113 outbreaks investigated in 2011 and 2012 were declared positive by a single result where weak amplification was observed (Additional file 1: Table S2). This supports the high false positivity rate observed during the clinical evaluation (Table 2). Previous studies have demonstrated that three specimens are ideal for the detection of norovirus outbreaks using RT-PCR, but excessive testing can lead to reduced specificity [5,40,41]. In this study, concurrent testing with multiple RT-PCRs was shown to increase the rate of false positive results, which could prematurely halt outbreak investigations caused by other enteric pathogens that might be managed differently [2].

## Conclusions

Unlike the high clinical sensitivity and specificity observed with real-time RT-PCR, this study demonstrated that end-point RT-PCRs had poor accuracy for the detection of circulating norovirus genotypes. To monitor norovirus epidemiology, genotyping should be considered part of routine outbreak investigations when norovirus is identified as the etiological agent. Unlike well established networks like Calicinet and Noronet, norovirus surveillance in Canada is in its infancy [2,14]. Ideally, sequencing would be used to encompass the regions required for genotyping and the primer/probe-binding sites for commonly used RT-PCRs [42,43]. When sequence mismatches are identified in RT-PCR target regions or when new norovirus variants emerges, proficiency panels should be promptly disseminated to clinical laboratories to ensure accurate detection [6]. With the dynamic nature of norovirus epidemiology, this study highlights the importance of routine surveillance and ongoing proficiency testing for circulating norovirus genotypes.

## Materials and methods

### Specimen preparation

For the clinical evaluation, 186 stool specimens were obtained from patients with acute gastroenteritis between March 15 and June 26, 2013. Public Health outbreak investigation data was provided by the Department of Health and Wellness (Halifax, NS) (Additional file 1: Table S2). Stool slurries were prepared by transferring 200 μl of stool into 500 μl of PCR-grade water and centrifugation (10,000 × *g*, 10 min). The supernatants (140 μl) were subjected to a total nucleic acid (TNA) extraction on a MagNA Pure LC instrument (Roche Diagnostics, Branchburg, NJ), as recommended by the manufacturer. TNAs were eluted in a volume of 60 μl and 5 μl served as template for all molecular assays (which were processed in parallel). Primers were synthesized by Sigma Genosys (Oakville, ON) and probes by Integrated DNA Technologies (Toronto, ON).

Analytical specificity was performed using high titer total nucleic acids extracted from norovirus stool suspensions of various genotypes obtained from collaborating laboratories and concentrated suspensions (MacFarlane value of 2.0) of various enteric pathogens that included: adenovirus type 40 (ATCC VR-931); *Camplobacter jejeuni* (ATCC 33291); *Clostridium difficile* (ATCC 9689); *Escherichia coli* O157:H7 (ATCC 35150); Rotavirus A (ATCC VR-2018); *Salmonella enterica* serovar Typhymirium (ATCC 14028); sapovirus (5 clinical isolates); *Shigella flexneri* (ATCC 12022); *Shigella dysenteriae* (ATCC 13313); *Yersinia enterocolitica* (ATCC 9610); *Vibrio cholerae* (clinical isolate). To assess the LoD, 10-fold serial dilutions of nucleic acids extracted from various norovirus genotypes were performed on specimens that had a minimum concentration of 10^6^ copies/ml. LoD was defined at a probability of 95% using Probit analysis [44] with replicate values obtained in three independent experiments (n = 9).

### End-point RT-PCRs

EP-JV and EP-SR RT-PCR assays were performed using a OneStep RT-PCR kit (Qiagen Inc., Mississauga, ON) in 50 μl reactions consisting of: 2 μl enzyme mix, 1× buffer, 400 μM dNTPs, 20 U RNaseOUT (Life Technologies, Burlington, ON), and 1.0 μM of each primer (JV12/JV13 for EP-JV or SR33/SR46 for EP-SR) (Additional file 2: Table S1). Amplifications were performed on a DNA Dyad Engine (Bio-Rad Laboratories Ltd., Mississauga, ON) as follows: 50°C for 30 min; 95°C for 15 min; and 40 cycles of 95°C for 1 min, 37°C (EP-JV) or 50°C (EP-SR) for 1 min, and 72°C for 1 min, followed by a final extension of 10 min at 72°C. Amplicons were resolved by 1% agarose gel electrophoresis with ethidium bromide staining. Expected sizes for EP-JV and EP-SR were 327 and 123 bp, respectively.

### Real-time RT-PCR

Real-time RT-PCR was performed in duplexed reactions by combining primers and probes commonly used for GI ad GII noroviruses (Additional file 2: Table S1). Briefly, PCR amplifications were performed on a Life Technologies ABI 7500 Fast instrument in 25 μl reactions consisting of: SuperScript III Platinum One-Step 1× master mix (Life Technologies), 0.2 μl enzyme mix, 20U RNaseOUT, and 400 nM of each primer and probe (Additional file 2: Table S1). Amplification conditions were as follows: 50°C for 30 min; 95°C for 30s; and 45 cycles of 95°C for 30s and 60°C for 1 min. Ct values were determined using the manufacturer's software (version 2.0.5).

### Discordant analysis and norovirus genotyping

Clinical sensitivity and specificity were calculated in comparison to a modified gold standard defined as concordant positive and negative results between real-time and end-point RT-PCRs (Table 2). Any discordant results were resolved by at the National Microbiology Laboratory (NML) using real-time RT-PCR with the same primers/probes but in monoplex reactions for GI and GII noroviruses (Additional file 2: Table S1). Since monoplex and duplex real-time RT-PCR targets were identical, a second reference method was also performed at the NML using RT-PCR amplification and sequencing of the norovirus major capsid protein regions C and D (Additional file 2: Table S1). The resulting sequence data was used for genotype assignment. Briefly, region C and D RT-PCR reactions were performed using a One-Step RT-PCR kit (Qiagen) in 50 μl reactions consisting of: 2 μl enzyme, 1× buffer, 400 nM dNTPs, 40 units of RNase Inhibitor, 10 μl of template, and 500 nM each primer (except CapB1 and CapD1 used at 1 μM) (Additional file 2: Table S1). Amplification conditions were as follows: 42°C for 30 min; 95°C for 15 min; 40 cycles of 94°C for 30s, 40°C (region D) or 50°C (region C) for 30s and 72°C for 30s; and a final extension of 72°C for 10 min. Following 2% agarose gel electrophoresis, amplicons were purified using Amicon Filter Devices (Millipore, USA) and sequencing was carried out by the Genomics Core section of the NML using primers CapA, CapB1, CapC, or CapD1.

### Norovirus genotyping and sequence alignments

BioNumerics 5.1 software (Applied Maths, Austin, TX) was used to assemble consensus sequence data, pairwise and global alignments, and clustering analysis. The sequences of each region were compared to ViroNet Canada reference dataset for genotype assignment.

To compare primer/probe binding sites to the target sequences of circulating norovirus genotypes (Figure 1), sequence data was retrieved from the Genbank database on the NCBI website (http://www.ncbi.nlm.nih.gov) and pairwise sequence alignments were performed using the Basic Local Alignment Search Tool (BLAST) function.

### Quantification of noroviruses

Norovirus genome equivalents were estimated in relation to a standard curve generated using plasmids harboring GI.4 (2008) and GII.4 (2006b) target sequences. RNA extracted from characterized stool specimens was used as template in 25 μl RT-PCR reactions consisting of: 1x One-Step RT-PCR buffer (Qiagen), 400 μM dNTPs, 0.4 μM of each primer (COG1F/COG1R for GI and COG2F/COG2R for GII) (Additional file 2: Table S1), 1 μl enzyme mix, and 4U RNaseOUT. Amplification conditions were as following: 42°C for 30 min; 95°C for 15 min; 45 cycles of 94°C for 30s, 50°C for 30s and 72°C for 1 min; and a final extension of 72°C for 5 min. Purified amplicons were cloned into pCR 2.1-TOPO vectors using a TOPO TA Cloning Kit according to manufacturer’s instructions (Life Technologies) and inserts were confirmed by DNA sequencing on a 3130XL Genetic Analyzer (Life Technologies) at Health Canada (Ottawa, ON). Following spectrophotometric quantification, 10-fold serial dilutions of the plasmids were used as template for the real-time RT-PCR. Ct values were plotted against plasmid concentration, generating inverse linear relationships [for GI (*y* = -3.44*x* + 42.13; R^2^ = 0.9995) and for GII (*y* = -3.53*x* + 41.63; R^2^ = 0.9998)]. Viral loads were expressed as log_10_ copies/ml and represent the average of triplicate values obtained in three independent experiments (n = 9).

### Statistical analysis

Chi-square and two-tailed Fisher's exact tests were used to compare proportions in 2-by-2 contingency tables. Binomial 95% confidence intervals and kappa statistics for each parameter were calculated by the "constant chi-square boundaries" method [45] using StatsPlus version 5.8.4.3 (AnalystSoft, Inc.).

## Competing interests

The authors declare that they have no competing interests.

## Authors’ contributions

BR and JL carried out the specimen processing and real-time and end-point RT-PCRs. JP performed the outbreak investigations. Discrepant analysis was performed by EG and TB. OM cloned the plasmids used for viral load determination. Specificity panels were prepared by XP. TB, TH, and JL were involved in the coordination and design of the study. All authors helped draft the manuscript and the final version was approved by all authors.

## Supplementary Material

Additional file 1: Table S2 Retrospective analysis of gastrointestinal outbreaks in Nova Scotia. Click here for file

Additional file 2: Table S1 Primer and probes used in this study [8-11,46,47]. Click here for file
