# Supplementary material for: Detection of circulating norovirus genotypes: hitting a moving target
Source: Virol J. 2014 Jul 18;11:129. doi: 10.1186/1743-422X-11-129 (PMC4112979; doi:10.1186/1743-422X-11-129)
Supplement: Additional file 1: Table S2 — Retrospective analysis of gastrointestinal outbreaks in Nova Scotia. [file 1743-422X-11-129-S1.doc]

**ADDITIONAL FILE 1**

Table S2. Retrospective analysis of gastrointestinal outbreaks in Nova Scotia.

| Time period | No. Specimens* | Avg. No. Specimens per Outbreak | % Pos.  Outbreaks | Proportion (%) of pos. outbreaks where the outcome was based on pos. results for both EP-JV and EP-SR** | Proportion (%) of positive outbreak where outcomes were based on a single weak positive result for either EP-JV or EP-SR*** | Estimated FPR for specimens submitted during an outbreak |
| --- | --- | --- | --- | --- | --- | --- |
| Jan. to Dec 2011 | 84 | 2.58  (1 to 7) | 62.9  (22/35) | 54.5  (12/22) | 18.2  (4/22) | 4.8  (4/84) |
| Jan. to Dec 2012 | 210 | 2.46  (1 to 12) | 74.4  (58/78) | 72.4  (42/58) | 23.8  (10/42) | 4.8  (10/210) |

* Number of specimens submitted as part of an outbreak investigation for norovirus RT-PCR. **This proportion is suggestive of true outbreaks. ***This data estimates the proportion of outbreaks that would be influenced by false positive results. Abbreviations: False positivity rate (FPR); number (No.); positive (Pos.).
