# Supplementary material for: Detection of circulating norovirus genotypes: hitting a moving target
Source: Virol J. 2014 Jul 18;11:129. doi: 10.1186/1743-422X-11-129 (PMC4112979; doi:10.1186/1743-422X-11-129)
Supplement: Additional file 2: Table S1 — Primer and probes used in this study [8-11,46,47]. [file 1743-422X-11-129-S2.doc]

**ADDITIONAL FILE 2**

Table S1. Primer and probes used in this study.

| Method | Name | Sequence (5’-3’) | Target | Reference |
| --- | --- | --- | --- | --- |
| EP-JV | JV12 | ATA CCA CTA TGA TGC AGA TTA | ORF1 (RdRp) | 11 |
|  | JV13 | TCA TCA TCA CCA TAG AAA GAG |  | 11 |
| EP-SR | SR33 | TGT CAC GAT CTC ATC ATC ACC | ORF1 (RdRp) | 8 |
|  | SR46 | TGG AAT TCC ATC GCC CAC TGG |  | 8 |
| Real-time  RT-PCR | COG1F | CGY TGG ATG CGI TTY CAT GA | ORF1-ORF2 junction | 9 |
|  | COG1R | CTT AGA CGC CAT CAT CAT TYA C |  | 9 |
|  | COG2F | CAR GAR BCN ATG TTY AGR TGG ATG AG |  | 9 |
|  | COG2R | TCG ACG CCA TCT TCA TTC ACA |  | 9 |
|  | Ring1A | FAM - AGA TYG CGA TCY CCT GTC CA - BHQ1 |  | 10 |
|  | Ring1B | FAM - AGA TCG CGG TCT CCT GTC CA - BHQ1 |  | 10 |
|  | Ring2 | FAM - TGG GAG GGC GAT CGC AAT CT - BHQ1 |  | 10 |
| NML | G1SKF | CTG CCC GAA TTY GTA AAT GA | ORF2 region C in norovirus GI | 46 |
|  | G1SKR | CCA ACC CAR CCA TTR TAC A |  | 46 |
|  | G2SKF | CNT GGG AGG GCG ATC GCA A | ORF2 region C in norovirus GII | 46 |
|  | G2SKR | CCR CCN GCA TRH CCR TTR TAC AT |  | 46 |
|  | Cap A | GGC WGT TCC CAC AGG CTT | ORF2 region D in norovirus GI | 47 |
|  | Cap B2 | TAT GTI GAY CCW GAC AC |  | 47 |
|  | CapB1 | TAT GTT GAC CCT GAT AC |  | 47 |
|  | CapC Rev | CCT TYC CAK WTC CCA YGG | ORF2 region D in norovirus GII | 47 |
|  | CapD3 Fwd | TGY CTY [I] T [I] CCH CAR GAA TGG |  | 47 |
|  | CapD1 Fwd | TGT CTR STC CCC CAG GAA TG |  | 47 |

Abbreviations: 6-carboxyfluorescein (FAM); black hole quencher (BHQ); open reading frame (ORF); National Microbiology Laboratory (NML); RNA-dependent RNA polymerase (RdRp); B = C, G, or T; H = A, C, or T; I = inosine; K = G or T; N = A, C, G, or T; R = A or G; S = C or G; W = A or T; Y = C or T.
